# In Vitro Propagation, Huperzine A Content and Antioxidant Activity of Three Genotypic *Huperzia serrata*

**DOI:** 10.3390/plants10061112

**Published:** 2021-05-31

**Authors:** Yan Yang, Liangfang Dai, Decai Wu, Limin Dong, Yisheng Tu, Jiankun Xie, Xiangdong Luo

**Affiliations:** College of Life Science, Jiangxi Normal University, Nanchang 330022, China; Yang0218yan@163.com (Y.Y.); libaibuhecha@163.com (D.W.); lyzong119@163.com (L.D.); ysttz2012@163.com (Y.T.); xiejiankun@jxnu.edu.cn (J.X.)

**Keywords:** genotypes, *Huperzia serrata*, Huperzine A, micropropagation, sterilization, antioxidant

## Abstract

*Huperzia serrata* is a traditional herb and endangered Chinese medicinal material, which has attracted much attention due to its production of Huperzine A (HupA). In vitro propagation of *H. serrata* is considered a new way to relieve the resource pressure of *H. serrata*. In this study, three different genotypic wild *H. serrata* were used for in vitro propagation. Then, the antioxidant activity and the content of HupA in the regenerated *H. serrata* were investigated. The results showed the survival rate of the explant was increased to 25.37% when using multiple sterilization processes. The best induction medium for *H. serrata* was the Schenk and Hildebrandt (SH) medium supplemented with 0.5 mg·L^−1^ Naphthalene acetic acid (NAA) and 0.1 mg·L^−1^ 2,4-Dichlorophenoxyacetic acid (2,4-D), where the regeneration rate of the explant was to 57.04%. The best proliferation medium was the SH medium with NAA (1.0 mg·L^−1^), as the biomass of in vitro tissue increased 164.17 ± 0.41 times. High-performance liquid chromatography analysis showed that the in vitro culture of three genotypes could produce HupA and the content of HupA was 53.90–87.17 µg·g^−1^. The antioxidant experiment showed that the methanol extract of in vitro *H. serrata* had higher antioxidant activity than that of wild *H. serrata*. This study provides a reliable in vitro *H. serrata* culture protocol and laid an important foundation for the antioxidant capacity of the thallus and the content of HupA.

## 1. Introduction

Alzheimer’s disease (AD) is a common neurodegenerative disease in aged population and has become the third leading cause of death after cardiovascular disease and cancer [1]. According to the world Alzheimer’s report of 2018, there are about 50 million AD patients in the world, and it will increase to 150 million by 2050. Currently, there are no effective drugs that can prevent AD pathogenesis or slow down its progression. Among the different therapeutic strategies of AD, an increase in the acetylcholine level in the brain using acetylcholinesterase inhibitors (AChEI) is considered to be an effective treatment to alleviate some of the symptoms of the disease [2]. A large number of clinical trials suggest that Huperzine A (HupA) demonstrates the favorable safety profiles at a relatively wide dose range and the potent efficacy in AD patients [3]. It has been confirmed that HupA is a highly effective, selective, and naturally reversible AChEI, with long-term action, ease in crossing the blood–brain barrier, and small side effects [4,5]. In China, HupA has been approved for the treatment of AD and vascular dementia, and phase III clinical trials have been completed in Europe [6]. So, it is a promising candidate for clinical development as a symptomatic treatment for AD [7,8].

*Huperzia serrata* (*H. serrata*) is a traditional Chinese herbal medicine used for the treatment of contusion, strain, swelling, and schizophrenia. Many *Lycopodium* alkaloids have been isolated from this plant. Since the highly effective AChEI Hup A was discovered in *H. serrata* [9], there has been more and more attention and research on *H. serrata* [10]. At present, *H. serrata*, the main source of HupA, actually possesses a very low content of HupA about 0.007% [11], grows very slowly, and finds it difficult to reproduce under natural conditions. It takes 6–15 years from spore germination to grow into a complete plant of about 12 cm high. For this reason, many researchers have tried to obtain HupA through chemical synthesis and endophytic fermentation [12,13,14].

In fact, the racemic synthesis of HupA has been achieved. However, regarding the in vitro inhibitory effects on the AChE enzyme, the racemic mixture of HupA is three times less potent than HupA from *H. serrata*. If used in large doses, contractions or other symptoms of cholinergic hyperactivity may occur [11], and this synthesis method requires more expensive reagents, such as phenyl selenium chloride, LDA, etc., which makes it difficult to meet the requirements of large-scale preparation [11]. Meanwhile, the fermentation yield of endophytic fungi is low and unstable. After several subcultures, the endophytic fungi could not continue to produce HupA [15,16]. Therefore, the production of HupA is still completely dependent on the wild *H. serrata* or its related species [10]. This has led to a large number of wild resources being widely harvested, and *H. serrata* was even at the risk of being endangered. So, the establishment of a suitable artificial propagation system is an important way to protect and utilize wild *H. serrata*.

Plant tissue culture is regarded as a powerful tool for micropropagation and conservation of unique plant germplasms. Studies on the micropropagation of *H. serrata* started in 1957, but the progress has been very slow because of the difficult sterilization and regeneration [17,18]. Recently, a few research groups have obtained in vitro thallus regenerated from *H. serrata* [19]. Previously, our group had also successfully obtained one genotypic thallus of *H. serrata,* which can produce HupA in vitro [20]. Then we further studied the correlation between HupA accumulation and the morphology of in vitro culture [21]. However, the contamination rate of *H. serrata* in previous studies is still relatively high and the experiment material of *H. serrata* is limited to one genotype. Meanwhile, previous studies on bioactive components and antioxidant activities of in vitro *H. serrata* are still very limited, which restricted the investigation and potential application of in vitro thallus.

Therefore, the regeneration and micropropagation system of different genotypic *H. serrata* was investigated in this study. In addition, the potential ability of HupA production and the antioxidant activity for different in vitro *H. serrata* were comparatively analyzed. These detailed results would be able to relieve the shortage of Chinese medicinal resources for HupA production and lay a very important foundation for germplasm improvement and genetic engineering studies for *H. serrata*.

## 2. Results

### 2.1. The Effect of Sterilization Methods on H. serrata Survival

**Figure 1:**
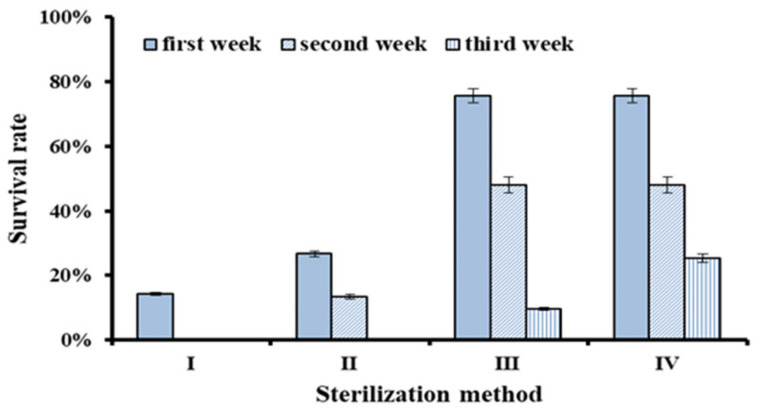
Explants survival rate of *H. serrata* for different sterilization method.

In order to improve the sterilization effect of *H. serrata* explants, different sterilization methods or combinations were used in this study (Table 1). The results showed that the survival rate of explants from different sterilization methods was significantly different (Figure 1). The survival rates in the first week were 14.29% for Method I, 26.67% for Method II, and 75.59% for Method III, respectively. Two weeks later, the survival rates were 0% (Method I), 13.33% (Method II), and 48.03% (Method III), respectively. Therefore, Method III was much better than Method II and I.

Two weeks after inoculation, about 50% of non-polluted explants of Method III were transferred to the regeneration medium containing antibiotics (Method IV). One week later, the survival rate of Method IV (25.37%) was higher than that of Method III (9.52%), which suggested that a certain concentration of antibiotics could inhibit endophytic fungi and would improve the survival rate of explants. Therefore, the best sterilization combination was 75% ethanol for 30 s, 10% hydrogen peroxide for 8 min, and 0.15% mercuric chloride for 5 min. After 2 weeks of regeneration culture, the non-polluted explants were transferred to a new regeneration medium containing 0.5 mg·L^−1^ malachite green and 100 mg·L^−1^ AAS (Antibiotic Antimycotic Solution, 100 mg AAS contains 50,000 IU of the penicillin, 50 mg streptomycin and 125 µg amphotericin B).

### 2.2. The Effects of Hormone Type and Concentration on H. serrata Regeneration

In order to assess the effect of hormone types and concentrations on the *H. serrata* regeneration, an orthogonal experiment was designed in the Table 2. Statistical analysis showed that the range corresponding to factor A (NAA) was the largest (23), followed by factor C (2,4-D) (17.98), and factor B (ZT) (13.70) was the smallest. These data indicated that NAA concentration (factor A) had a significant difference on the thallus regeneration of *H. serrata,* followed by 2,4-D (factor C).

Further analysis showed that there are significant differences among the sum of test index (induction rate) corresponding to different levels of each factor. For NAA (factor A), the sum of induction rate reaches a maximum at 0.5 mg/L (Level 2 of factor A), that is KA_2_ > KA_3_ > KA_1_ (Table 1). For factor B (ZT) and C (2,4-D), the maximum value of the sum of induction rate was at 0 mg·L^−1^ (Level 1 of factor B) and 0.1 mg·L^−1^ (Level 2 of factor C), respectively. So, the most suitable hormone combination for *H. serrata* regeneration was A_2_B_1_C_2_. That is to say, the best induction medium for *H. serrata* regeneration was SH medium supplemented with 0.5 mg·L^−1^ NAA and 0.1 mg·L^−1^ 2,4-D [22,23], whereby the regeneration rate of the *H. serrata* explant was increased to 57.04% (Table 2).

### 2.3. The Effects of Hormone Type and Concentration on the H. serrata Proliferation

**Figure 2:**
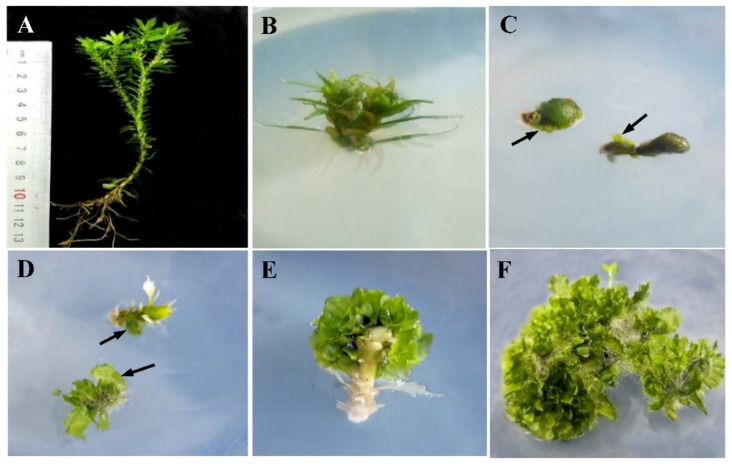
In vitro micropropagation process of *H. serrata*. (**A**) Wild plant of Fujian genotype. (**B**) Inoculated stem. (**C**) Regeneration from callus, arrows shows the regenerated thallus. (**D**,**E**) Thallus regeneration from spores on stems. (**F**) Thallus proliferation.

In order to investigate the effect of hormone types and concentrations on in vitro *H. serrata* proliferation, a multi-factor randomized comparative test was designed. As shown in Table 3, the effects of different hormone concentrations and types were significantly different (*p* < 0.05). The relative growth rate of the treatment group (supplemented with hormones group) was significantly higher than that of the control group (No hormones group). When the SH medium was supplemented with 1.0 mg·L^−1^ NAA, the relative growth rate achieved the maximum value (164.17 ± 0.41 times), which was about 2 times that of the control group. When the NAA concentration was at 1.5 mg·L^−1^, the relative growth rate of in vitro *H. serrata* reduced to 114.88 ± 2.13 times, which indicated that the excessive hormone would inhibit the in vitro *H. serrata* proliferation. A similar trend was observed among the IAA treatment. The corresponding relative growth rate was less than that of the NAA treatment group with the same concentration. So, the best multiplication medium was SH medium with NAA 1.0 mg·L^−1^ for *H. serrata* proliferation. The process of in vitro propagation of *H. serrata* is shown in Figure 2.

### 2.4. Morphological Difference of Different H. serrata Thallus

**Figure 3:**
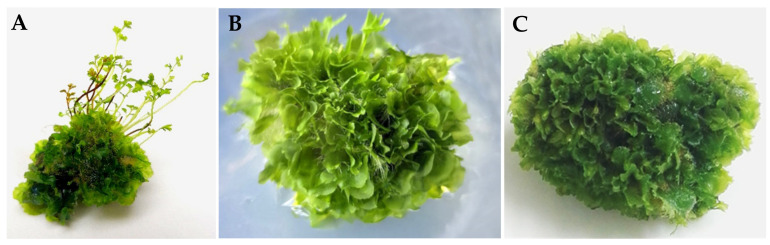
Morphological difference of three different genotypic *H. serrata*. (**A**) HB. (**B**) FJ. (**C**) JX.

In this study, some morphology and growth differences of the three genotypes in vitro *H. serrata* thallus were observed. As shown in Table 4, the thallus of the HB genotype grew more rapidly. A 164.17 ± 0.41-fold increase in biomass has increased, followed by the FJ genotype (153.71 ± 0.53 times) and the JX genotype (121.08 ± 1.09 times). For the morphological difference of thallus, the thallus of HB was thin and long, FJ’s were wide and large, and JX’s were thin and short (Table 4, Figure 3). As for the size of the thallus, the diameter of HB and JX were 1.74 ± 0.73 cm and 1.18 ± 0.15 cm, respectively, while that of FJ was 2.59 ± 0.21 cm. As shown in Figure 3, the morphology of the thallus for HB and JX genotype was compact, while that of FJ was loose. In addition, there were some minor differences among the three genotypic in vitro *H. serrata*, including the regeneration rate and other morphological characteristics. These results indicated that the genotypes had some effect on micropropagation in vitro *H. serrata*.

### 2.5. HupA Detection in Thallus of H. serrata

**Figure 4:**
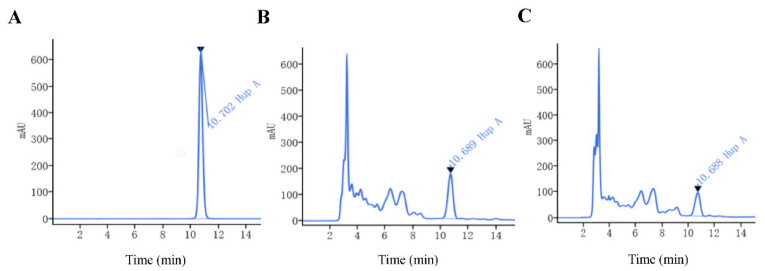
Chromatogram of the Huperzine A in different sample. (**A**) Chromatogram of the HupA standard. The retention time for HupA standard was 10.702 min. (**B**) Sample chromatogram of *H. serrata* extract (plant collected in Fujian Table 5). The retention time for the HupA was 10.689 min. (**C**) Sample chromatogram of the FJ’ propagated extract (plant collected In vitro). The retention time for the HupA was 10.688 min.

In vitro propagation of the plant material that produces HupA has attracted attention in recent years. One of the purposes of this study is to evaluate the ability of HupA production in different genotypic in vitro *H. serrata* thallus. HPLC analysis suggested that HupA content of the HB genotype was the highest among the three wild *H. serrata*, which was 220.34 ± 3.08 µg·g^−1^ (Table 5). The HupA content of wild *H. serrata* from FJ and JX was 198.12 ± 1.71 µg·g^−1^ and 186.38 ± 1.84 µg·g^−1^, respectively. The retention time for the HupA standard was 10.702 min (Figure 4A). The chromatogram of the wild FJ genotype is shown in Figure 4B, and the retention time was 10.682 min.

For the thallus, the HupA content of FJ genotype was the highest, which was 87.17 ± 5.15 µg·g^−1^, and in the thallus of the JX and HB genotypes, the HupA content was 76.28 ± 4.26 µg·g^−1^ and 53.90 ± 3.96 µg·g^−1^, respectively. The chromatogram of FJ’s thallus is shown in Figure 4C. The content of HupA in the thallus was about one-third of that in the corresponding wild *H. serrata*. These results indicated that genotype and environment would significantly affect the accumulation of HupA in *H. serrata*. Besides, thallus grew rapidly and was not limited by seasons. The accumulation of HupA in per-unit time was faster than that of wild *H. serrata*.

The linear relationship between the peak area (y) and concentration (x, µg·mL^−1^) of the HupA standard is represented by the regression equation (y = 18182x − 14.312). The calibration curve of this compound shows good linearity (R^2^ = 1.0000).

### 2.6. The Antioxidant Activity Analysis

**Figure 5:**
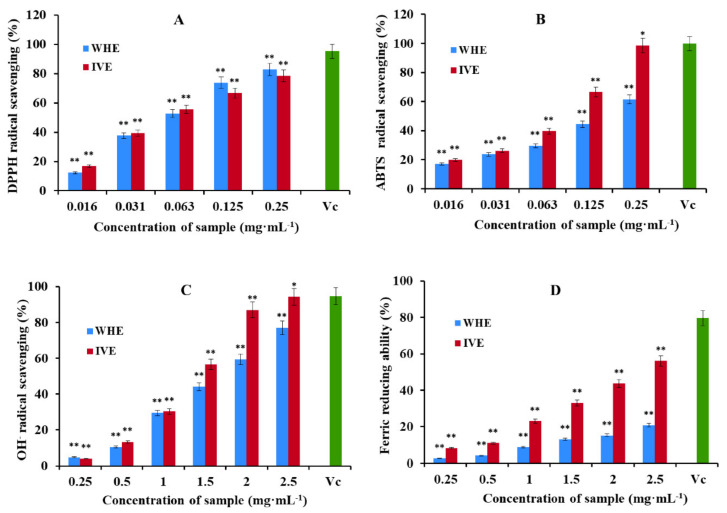
(**A**) DPPH scavenging capacity of the methanol extract of the FJ’s wild *H. serrata* (WHE) and the methanol extract of the in vitro (IVE), Vitamin C (Vc, 0.010 mg/mL) was used as a positive control. (**B**) ABTS scavenging capacity, Vc (0.25 mg/mL) was used as a positive control. (**C**) OH^−^ scavenging capacity, Vc (2.5 mg/mL) was used as a positive control. (**D**) Ferric reducing ability, Vc (2.5 mg/mL) was used as a positive control. All measurements are expressed as means ± SD of three separate determinations. * *p* < 0.05 and ** *p* < 0.01 vs. control.

Figure 5 shows the relationship between antioxidant activity and different concentrations of samples in the form of a bar graph. DPPH and ABTS radical scavenging activity is shown in Figure 5A,B. The results showed that the DPPH and ABTS radical scavenging activity of the in vitro *H. serrata* methanol extract (IVE) was significantly greater than that of the wild *H. serrata* methanol extract (WHE). The highest scavenging activity of IVE was 82.90 ± 0.56% for DPPH and 98.64 ± 1.00% for ABTS at the concentration of 0.25 mg·mL^−1^. However, the DPPH and ABTS radical scavenging activity of WHE was 78.50 ± 0.45% and 61.59 ± 0.68%, respectively. IC50 values of IVE in DPPH and ABTS scavenging assays were 0.051 and 0.061 mg·mL^−1^, respectively. IC50 values of WHE in DPPH and ABTS scavenging assays were 0.064 and 0.155 mg·mL^−1^, respectively (Table 6).

The OH^−^ scavenging activity and the ability to reduce Fe^3+^ of WHE and IVE are shown in Figure 5C,D. The results showed that the hydroxyl radical scavenging activity and the ability to reduce Fe^3+^ of IVE was significantly greater than that of WHE at the same tested concentration. The highest scavenging activity of IVE was to 94.29 ± 1.02% for OH^−^ scavenging capacity and 56.07 ± 0.61% for the ability to reduce Fe^3+^ value at the concentration of 2.5 mg·mL^−1^. However, it is 77.05 ± 0.72% and 20.77 ± 0.84% in WHE, respectively. IC50 values of IVE in OH^−^ scavenging activity and the ability to reduce Fe^3+^ were 1.37 and 2.52 mg·mL^−1^, respectively. IC50 values of WHE in OH^−^ scavenging activity and the ability to reduce Fe^3+^ were 1.67 and 9.73 mg·mL^−1^, respectively (Table 6).

The antioxidant capacity of plants is related to polyphenol compounds. Therefore, experiments were designed to detect the total polyphenol content of WHE and IVE. The linear relationship between the absorbance (y) and concentration (x, µg·mL^−1^) of the gallic acid standard is expressed by the regression equation y = 6.8x + 0.0597, R^2^ = 0.9935. The total polyphenol content of WHE was 12.97 ± 0.81 mgGAE/g. The total polyphenol content of IVE is 14.96 ± 0.65 mgGAE/g.

From the experiment of the antioxidant, it shows that in vitro *H. serrata* has a strong antioxidant effect. The extract of in vitro *H. serrata* had an excellent scavenging effect on DPPH, ABTS, and OH^−^ and obviously had the ability to reduce Fe^3+^. Through the detection of total polyphenol content, micropropagation increased the antioxidant capacity by increasing the content of polyphenol compounds.

## 3. Discussion

*H. serrata* is an ancient fern plant group with important medicinal value. The fast-growing demand and the high price of the raw material are increasing the pressure on natural habitats, and *H. serrata* has become a threatened plant in China due to the over-exploitation and habitat fragmentation. Furthermore, besides not being particularly abundant in wild resources, these plants also grow extremely slowly [24,25]. Thus, owing to the unique bioactivity of HupA and its low yield from plants, several research groups have devoted intensive efforts to study the in vitro culture of *H. serrata*. However, the progress of these studies has been very slow and cannot be commercialized on a large scale. In order to alleviate the bottleneck of HupA production, it is of great significance to establish an in vitro rapid propagation system for *H. serrata*, which would be very important for the sustainable development and utilization of *H. serrata*.

Rapid propagation in vitro has the advantages of short growth cycle, high reproduction rate, and artificial control of culture conditions. One of the important reasons for slow progress is that *H. serrata* is rich in endophytic fungi, which makes sterilization difficult [26]. Moreover, the cuticle of *H. serrata* leaves is thin, and it is easy to be killed by excessively disinfection. Therefore, the effective sterilization method is the key step for micropropagation of *H. serrata*. Some researchers have carried out a series of research and investigations on the sterilization methods for *H. serrata* explants [27,28]. Shen et al. used *H serrata* stems as explants for tissue culture and found that it was very difficult to sterilize although it was sterilized several times [29]. Szypula et al. found that the antibiotics would be helpful for removing the endophytic fungi of the explant [30]. However, the survival rate of explants in previous studies was still very low. In this study, multiple sterilization methods were used to sterilize *H. serrata* explants. Compared with previous studies, the survival rate of surviving explants without obvious bacterial and endophyte infection after three weeks was significantly improved, which was 25.37%. These results laid an important foundation for the subsequent induction and regeneration of *H. serrata*.

During the past years, there were some successful reports on in vitro propagation of *H. serrata* and its similar species. Szypula et al. selected the suitable medium for *H. selago* explants surviving and growing [30]. Ma and Gang succeeded in propagating *Phlegmariurus squarrosus* (Forst.) in vitro and in detecting HupA produced by the corresponding cultivated plant [18]. Shoot tips of *H. pinifolia* were induced into callus, producing HupA [31]. We also obtained HupA-producing *H. serrata* thallus through in vitro culture [32]. However, previous regeneration was limited to one genotype, and the micropropagation efficiency is not high. In the present study, an orthogonal experiment and mult-factor experiment were used to establish the in vitro rapid propagation system of *H. serrata*. Three different genotypic thallus of *H. serrata* in vitro were obtained, of which the regeneration rate (57.04%) and the biomass increased 164.17 ± 0.41 times. In addition, HPLC detection of its thallus could produce HupA. These results are of great significance for solving the resource shortage of *H. serrata* for HupA production.

Previous studies suggested that the environmental conditions and genotype would play important roles in controlling HupA production in *Huperzia* species [33]. Ma et al. detected *H. serrata* in Yunnan, Hunan, and Sichuan, and the content of HupA in its body was 148 µg·g^−1^, 80.2 µg·g^−1^, and 182.6 µg·g^−1^ respectively [9,31]. Li et al. found that in *H. serrata* from the Hunan Province (Baiyun Mountain), the Fujian Province (Nanping Municipal county), and the Jiangxi Province (Yifeng county), the levels of HupA are 233.1 µg·g^−1^, 148.9 µg·g^−1^, and 321.7 µg·g^−1^, respectively [34]. The results of this study showed that the content of HupA in the three different genotypes was significantly different. As for the content of Hup A in cultured tissue, Bao et al. used HPLC to determine the content of HupA in *H. serrata* tissue culture and wild sporophytes. The results showed that the content of HupA in cultured tissue was one-fourth of that in wild sporophytes [19]. Our experimental results show that the HupA content of in vitro *H. serrata* was about one-third of that of corresponding wild *H. serrata*. We also found that the content of HupA could be increased by adding some metabolic substrates during the proliferation culture [35,36]. Since the in vitro *H. serrata* thallus grew rapidly and was not limited by seasons, this in vitro propagation system of *H. serrata* would be able to relieve the shortage of Chinese medicinal resources for HupA production.

Previous studies have shown that micropropagation exhibited a remarkable influence on phytochemical contents and morphology of plants. Plants that undergo micropropagation have higher contents of phenolics and flavonoids and antioxidant activity compared to those developed from the wild [37]. The phenols and flavonoids are related to the antioxidant properties of plants. Our results of this study found that the antioxidant activity of thallus was stronger than that of wild *H. serrata*, indicating that the advantages of micropropagation of thallus to produce antioxidant metabolites makes it available to fulfill the high pharmaceutical demands. It opens up the practical application value of these rapidly propagating plants. The increased antioxidant capacity of the in vitro *H. serrata* may be related to the culture environment and the enrichment of the culture medium during the propagation period. Plant growth regulators contained in micropropagation promote the biosynthesis of antioxidant-related compounds by influencing the expression or up-regulation of genes in the biosynthetic pathway of secondary metabolites.

## 4. Materials and Methods

### 4.1. Plant Material

The plant materials of *H. serrata* used in this study were collected from three different provinces in China during June and November 2018. The locations of the different genotypic *H. serrata* were shown in the Table 7. The samples were authenticated by Professor ZR Zou, a botanist of Jiangxi Normal University. Then, the fresh spore-bearing stems of *H. serrata* from the three areas were used for the subsequent micropropagation in vitro, and the remaining plant of *H. serrata* was freeze-dried and stored for HupA detection and antioxidant activity analysis.

### 4.2. Sterilization of the Explants

The explants that were stem segments with stem tips and spores were cut into a length of 1–3 cm and excess leaves were removed. Firstly, the explants were rinsed 3 times with sterile water and were put on the super-clean worktable. Then the explants were sterilized by various sterilization combinations (Method I–III), which is shown in Table 1. After surface disinfection, the explants were rinsed with sterile water for 3–4 times. Then the sterilized explants were used for inoculation on the regeneration culture medium. The explants survival rate was counted every 7 d. Each treatment was conducted in 10 bottles; three explants were inoculated in each bottle and repeated 3 times. This is example 1 of an equation:

Survival rate = the number of viable explants/the number of explants in the primary medium × 100%.(1)

In order to investigate the removal effect of antibiotics on endophytic fungi, we developed another explants sterilization method (Method IV), which was based on the Method III (Table 1). More concretely, after a 2-week regeneration culture of Method III, some non-polluted explants were transferred to a new regeneration medium, which was supplemented with 100 mg/L AAS (Antibiotic Antimycotic Solution, there were 50,000 IU of the penicillin, 50 mg streptomycin and 125 µg amphotericin B per 100 mg AAS) and 0.5 mg·L^−1^ malachite green, and the other components of the regeneration medium remained unchanged (Table 2).

### 4.3. Regeneration Culture

The method of orthogonal design was used for regeneration culture, which was used to investigate the effects of hormone types and concentrations on regeneration induction (Table 2). The basic medium of regeneration culture was the SH medium with agar 6.5 g·L^−1^, sucrose 20 g·L^−1^, and pH 5.8–6.0. In addition, the different regeneration medium was supplemented with a different growth regulator, which is listed in Table 3. The regeneration culture experiment adopted a 3-factor 3-level orthogonal design L_9_ (3^4^) (Table 2), and each treatment was conducted in 10 bottles; three explants were inoculated in each bottle and repeated 3 times. The culture room was maintained at 25 ± 1 °C. The photoperiod was 2000 lx for 13 h day light, 11 h darkness. The explants were incubated for 6–8 weeks under the culture condition, and the regeneration rate was counted.

Regeneration rate = regenerated plants/surviving plants × 100%.(2)

### 4.4. Proliferation Culture

After 40–60 days, the actively growing thallus of *H. serrata* in the regeneration medium was used for proliferation culture. About 0.5 × 0.5 cm thallus were transferred to the proliferation medium with a different growth regulator for 60–80 d (detailed in Table 3). The composition of the basic medium and culture conditions were the same as that of the regeneration culture. The initial weight was 17.33 ± 1.15 mg. Each treatment was repeated 3 times.

Biomass growth times = (plant fresh weight after 60 days/initial weight).(3)

### 4.5. HupA Content Analysis

HupA was extracted from in vitro *H. serrata* thallus and wild *H. serrata* referring to previous reports with some modifications [26,38]. Specifically, the vigor thallus was collected after proliferation culture for 80 d, and the thallus and its corresponding wild plants were dried under low temperature. The dried samples of 0.5 g each of powdered plant material were extracted with 2% tartaric acid for 24 h in a water bath at 54 °C. Then the filtrate was extracted three times by an ultrasonic bath for 30 min. The combined filtrates were evaporated to dry powder, dissolved in methanol (HPLC purity grade), and passed through a 0.22 μm Millipore poly (tetrafluoroethylene) (PTFE, 0.22 μm) syringe filter into a 2.0 mL glass vial and adjusted to volume for HPLC analysis. The purity of HupA analytical standard was ≥98.0%, purchased from Aladdin Industrial Corporation (Shanghai, China). The HupA analytical standard was weighed and dissolved in methanol at 1.0 mg·mL^−1^. The stock solutions were diluted with methanol to yield a series of standard solutions for use in quantitative analyses.

### 4.6. HPLC Conditions and Equipment

High-performance liquid chromatography (HPLC) analyses were performed on the Agilent 1260 mode (Agilent Technologies, Palo Alto, CA, USA) system consisting of a quaternary pump, an integrated diode-array detector, and an automated sample injector and data system. The separation of *H. serrata* alkaloids was performed on the EC 250/4.6 Nucleosil1 120–7 mm C18 column. The eluent was a mixture of methanol: ammonium acetate (0.08 mol·L^−1^, pH 6.00) (30:70). The flow rate was set at 0.8 mL·min^−1^, and eluent was monitored at 308 nm. HupA was used as a standard substance. All eluents were of HPLC purity grade.

### 4.7. Antioxidant Activity Analysis

The dried samples of *H. serrata* (10 g) were extracted three times with methanol (150 mL) at room temperature. The extracted solution was filtered through filter paper and concentrated using a rotary evaporator under a vacuum to obtain a powdered extract. The extract was dissolved in methanol at a concentration of 10 mg·mL^−1^ and filtered through a syringe filter (0.45 µm) for quantitative analysis. The Vc was weighed and dissolved in methanol at 10 mg·mL^−1^. The stock solutions were diluted with methanol to yield a series of standard solutions for use in quantitative analyses. The wild-type material was from Fujian, and the tissue culture type was thallus cultured in vitro from the wild-type material from Fujian. The purity of gallic acid was ≥99.0%, purchased from Aladdin Industrial Corporation (Shanghai, China).

The determination of antioxidant activity of the wild *H. serrata* methanol extract (WHE) and in vitro *H. serrata* methanol extract (IVE) was evaluated using the method described by Zhang et al. [39] and Wan et al. [40] with minor modifications. The antioxidant experiments include DPPH, ABTS, and OH^−^ radical scavenging activity and the ability to reduce Fe^3+^. The DPPH and ABTS radical scavenging capacities of the tested samples were calculated using the following Equation (4). The OH^−^ radical scavenging rate and the ability to reduce Fe^3+^ of the tested samples were calculated using the following Equation (5).

Scavenging rate (%) = 1 − (A_i_ − A_j_)/A_o_ × 100% (4)

Scavenging rate (%) = (A_i_ − A_o_)/(A_j_ − A_o_) × 100% (5)

where A_o_ is the absorbance of control, A_i_ is the absorbance of samples, and A_j_ represents reagent blank absorbance. Each concentration was done in triplicate, and a positive control (Vc) was treated under the same conditions as the samples.

### 4.8. Statistical Analysis

All measurements are expressed as means ± SD of three separate determinations. The effect of different treatments was analyzed using one-way analysis of variance (ANOVA) and the difference between their means was compared using Fisher’s least significant difference (LSD) test at 5% *p*-value threshold. All the analyses were carried out using SPSS 25.0.

## 5. Conclusions

This study showed that the key to establish an in vitro propagation system of *H. serrata* is the sterilization of explants. It is confirmed that the multiple sterilization method can achieve the effect of effective sterilization. This study focused on how to establish the in vitro propagation system of three genotypes of *H. serrata* and determined, in detail, the antioxidant activity and the content of HupA for in vitro *H. serrata*. Thus, the present protocol may be used to produce plants to meet the gap of demand, supply, and conservation.

## Figures and Tables

**Table 1:** Different sterilization combination or process of *H. serrata*.

Method	75% Alcohol (s)	10% Hydrogen Peroxide (min)	0.15% Mercuric Chloride (min)	AAS (mg·L^−1^)	Malachite Green (mg·L^−1^)
Method I	30	0	5	0	0
Method II	30	8	0	0	0
Method III	30	8	5	0	0
Method IV	30	8	5	100	0.5

**Table 2:** Modification of medium used to induction culture *H. serrata*.

Number	Factor			Regeneration Rate (%)
	NAA (Factor A)	ZT (Factor B)	2,4-D (Factor C)	
1	0	0	0	0
2	0	0.2	0.1	15.08
3	0	1	0.2	10.74
4	0.5	0	0.1	57.04
5	0.5	0.2	0.2	23.81
6	0.5	1	0	13.99
7	1.0	0	0.2	26.41
8	1.0	0.2	0	21.83
9	1.0	1	0.1	17.63
K_j1_	25.82	83.45	35.82	
K_j2_	94.84	60.72	89.75	
K_j3_	65.87	42.36	60.96	
range	23	13.70	17.98	
Primary and secondary order	A > C > B			
Optimal combination	A_2_B_1_C_2_			

Note: Kj_1_ is the sum of test index (induction rate) corresponding to the factor 1 level of column j, Kj_2_ is the sum of test index corresponding to the factor 2 level of column j, Kj_3_ is the sum of test index corresponding to factor 3 level of column j, and j represents factor A, B or C.

**Table 3:** Modification of medium used to proliferation culture *H. serrata*.

Growth Additive	Concentration (mg·L^−1^)	Biomass Growth Times	*p*-Value
Control group	0	83.61 ± 1.22	*p* < 0.05
NAA	0.5	117.58 ± 1.52	
	1.0	164.17 ± 0.41	
	1.5	114.88 ± 2.13	
IAA	0.5	97.75 ± 1.69	
	1.0	142.58 ± 1.33	
	1.5	110.33 ± 1.60	

Note: Biomass growth times = (plant fresh weight after 60 days/initial weight), the initial weight is 17.33 ± 1.15 mg Data are presented as means ± SD of three independent experiments. Each treatment group is significantly different at the 0.05 level (LSD) compare to control group.

**Table 4:** Growth status of three genotypic *H. serrata*.

Genotypes	Biomass Growth Times	Morphological Difference	Tissue Diameter (cm)
HB	164.17 ± 0.41	tenuous	1.74 ± 0.73 b
FJ	153.71 ± 0.53	wide	2.59 ± 0.21 a
JX	121.08 ± 1.09	tiny	1.18 ± 0.15 c

Note: Data are presented as means ± SD of three independent experiments. Different lowercase letters indicate that LSD is significantly different at the 0.05 level between each group.

**Table 5:** Hup A content in different *H. serrata*.

Genotypes	Wild *H. serrata* (µg·g^−1^)	Thallus In Vitro (µg·g^−1^)
HB	220.34 ± 3.08 a	53.90 ± 3.96 c
FJ	198.12 ± 1.71 b	87.17 ± 5.15 a
JX	186.38 ± 1.84 c	76.28 ± 4.26 b

Note: Data are presented as means ± SD of three independent experiments, different letters show differences between groups *p* < 0.05.

**Table 6:** The value of IC50.

IC50	Vc (mg·mL^−1^)	WHE (mg·mL^−1^)	IVE (mg·mL^−1^)
DPPH	0.005	0.064	0.051
ABTS	0.017	0.155	0.061
OH^−^	0.068	1.670	1.370
Fe^3+^	0.035	9.730	2.520

**Table 7:** Explants of three different genotypic *H. serrata* used for micropropagation In vitro.

Genotype Code	Location	Longitude (E)/Latitude (N)	Altitude (m)	Habit
FJ	Wuyi Mount, Fujian province	117°24′/27°32′	350–2518	Humid primary forest
HB	Wuling Mount, Hubei province	111°68′/29°03′	1000–2572	Secondary forest shade
JX	Lushan, Jiangxi province	115°48′/29°36′	1000–1473	Primary forest close to ridge

## Data Availability

All the data are included in the present study.

## References

[1] Bienaimé C., Aurélie M., Lamine B., Jacques A., Edmundo N.S., Sylvie B.R. (2015). Effects of plant growth regulators on cell growth and alkaloids production by cell cultures of *Lycopodiella inundata*. Plant Cell Tissue Organ Cult..

[2] Kushal K., Kumara A., Keeganc R.M., Deshmukha R. (2018). Recent advances in the neurobiology and neuropharmacology of Alzheimer’s disease. Biomed. Pharmacother..

[3] Lin P.P., Li X.N., Yuan F., Chen W.L., Yang M.J., Xu H.R. (2016). Evaluation of the in vitro and in vivo metabolic pathway and cytochrome P450 inhibition/induction profile of Huperzine A. Biophys. Res. Commun..

[4] Jiang W.W., Liu F., Gao X., He J., Cheng X., Peng L.Y., Wu X.D., Zhao Q.S. (2014). Huperserines A-E, *Lycopodium* alkaloids from *Huperzia serrata*. Fitoterapia.

[5] Wu S.L., Gan J., Rao J., He S.J., Zhu W.W., Zhao Y., Lv Y.N., Huang J.G., Liu Y.N. (2017). Pharmacokinetics and tolerability of oral dosage forms of Huperzine A in healthy Chinese male volunteers: A randomized, single dose, three-period, six-sequence crossover study. Curr. Med. Sci..

[6] Xie L.S., Jiang C., Wang Z., Yi X.H., Gong Y.Y., Chen Y.H., Fu Y. (2016). Effect of Huperzine A on Aβ-induced p65 of astrocyte in vitro. Bull. Agric. Chem. Soc. Jpn..

[7] Ma X.Q., Tan C.H., Zhu D.Y., Gang D.R., Xiao P.G. (2007). Huperzine A from Huperzia species—An ethnopharmacolgical review. J. Ethnopharmacol..

[8] Xiong Z.Q., Yang Y.Y., Liu Q.X., Sun C.C., Jin Y., Wang Y. (2015). Endophytes in the plant *Huperzia serrata*: Fungal diversity and discovery of a new pentapeptide. Arch. Microbiol..

[9] Ma X.Q., Tan C.H., Zhu D.Y., Gang D.R. (2005). Is there a better source of Huperzine A than *Huperzia serrata*? Huperzine A content of Huperziaceae species in China. J. Agric. Food Chem..

[10] Guo B., Ren J.Y., He M.N., Yao K., Wang T.S., Wang L.Q., Liu X., He W., Fu Y.P., Wang D.L. (2019). Development of polymorphic simple sequence repeat marker in *Huperzia serrata* (Lycopodiaceae). Appl. Plant Sci..

[11] Ferreira A., Rodrigues M., Fortuna A., Falcão A., Alves G. (2016). Huperzine A from *Huperzia serrata*: A review of its sources, chemistry, pharmacology and toxicology. Phytochem. Rev..

[12] Cruz-Miranda O.L., Folch-Mallol J., Martínez-Morales F., Gesto-Borroto R., Villarreal M.L., Taketa A.C. (2020). Identification of a Huperzine A-producing endophytic fungus from *Phlegmariurus taxifolius*. Mol. Biol. Rep..

[13] Jiang F.F., Qi B.W., Ding N., Yang H.Y., Jia F.F., Luo Y., Wang J., Liu X., Wang X.H., Tu P.F. (2019). *Lycopodium* alkaloids from *Huperzia serrata*. Fitoterapia.

[14] Zhan Z.J., Tian T., Xu Y.L., Yu H.F. (2019). Biotransformation of Huperzine B by a fungal endophyte of *Huperzia serrata*. Chem. Biodivers..

[15] Yang H.L., Peng S.L., Zhang Z.B., Yan R.M., Wang Y., Zhan J.X., Zhu D. (2016). Molecular cloning, expression, and functional analysis of the copper amine oxidase gene in the endophytic fungus Shiraia sp. Slf14 from *Huperzia serrata*. Protein Expr. Purif..

[16] Zhu D., Wang J., Zeng Q., Zhang Z., Yan R. (2010). A novel endophytic Huperzine A–producing fungus, Shiraia sp. Slf14, isolated from *Huperzia serrata*. J. Appl. Microbiol..

[17] Freeberg J.A., Wetmore R.H. (1957). Gametophytes of *Lycopodium* as grown in vitro. Phytomorphology.

[18] Ma X.Q., Gang D.R. (2008). In vitro production of Huperzine A, a promising drug candidate for Alzheimer’s disease. Phytochemistry.

[19] Bao R.S., Yin P.P., Guo B., Wei Y.H. (2012). Prothallium culture and sporophyte induction of *Huperzia serrata* (Thunb.) Trev. Acta Phytophysiol..

[20] Ji Z.D., Tu Y.S., Ding M.H., Chen X., Jiang X.F. (2014). In vitro culture of *Huperzia serrata* thallus for the medicinal component production. Nat. Prod. Res. Dev..

[21] Xu X.Z., Tu Y.S., Ji Z.D., Chen M., Cai X.F., Yang P. (2015). Study on the morphological changes of *Huperzia serrata* in vitro culture and the accumulation of Huperzine A. Chin. Bull. Bot..

[22] Schenk R.U., Hildebrandt A.C. (1972). Medium and techniques for induction and growth of monocotyledonous and dicotyledonous plant cell cultures. Can. J. Bot..

[23] Zahir A., Ahmad W., Nadeem M., Giglioli-Guivarc’h N., Hano C., Abbasi B.H. (2018). In vitro cultures of *Linum usitatissimum* L.: Synergistic effects of mineral nutrients and photoperiod regimes on growth and biosynthesis of lignans and neolignans. J. Photochem. Photobiol. B.

[24] Huang J., He C.Z. (2010). Population structure and genetic diversity of *Huperzia serrata* (Huperziaceae) based on amplified fragment length polymorphism (AFLP) markers. Biochem. Syst. Ecol..

[25] Qi Y.D., Wang D.L. (2017). Population structure and resource reducing factors of *Huperzia serrata* (Thunb.ex Murray) Trev. in China. Chin. Mod. Med..

[26] Lim W.H., Goodger J.Q.D., Field A.R., Holtum J.A.M., Woodrow I.E. (2010). Huperzine alkaloids from Australasian and southeast Asian *Huperzia*. Pharm. Biol..

[27] Ma Y.Z., Liu J.H., Xu H., Liu F. (2015). In vitro culture of *Huperzia serrata*. J. Plant Physiol..

[28] Zhang X.H., Li X.J., Yang X.F., Luo J.P. (2016). Effects of different conditions on the growth of micropropagated *Huperzia serrata* plantlets and the accumulation of Huperzine. Chin. Mod. Tradit Med..

[29] Shen X.X., Yu X.P., Sheng S.J. (2002). Study on the method of tissue culture and sterilization of the stem tip of Melaleuca pagoda. Chin. J. Chin. Mater. Med..

[30] Szypuła W., Pietrosiuk A., Suchocki P., Olszowska O., Furmanowa M., Kazimierska O. (2005). Somatic embryogenesis and in vitro culture of *Huperzia selago* shoots as a potential source of Huperzine A. Plant Sci..

[31] Ishiuchi K., Park J.J., Long R.M., Gang D.R. (2013). Production of Huperzine A and other *Lycopodium* alkaloids in *Huperzia* species grown under controlled conditions and in vitro. Phytochemistry.

[32] Yuan H.H., Tu Y.S., Huang Q., Yu X. (2019). Studies on Huperzine A accumulation and biochemical characteristics in thallus of *Huperzia serrata* in vitro by liquid culture. J. Trop. Subtrop. Bot..

[33] Xu M.N., Heidmarsson S., Boer H.J.D., Kool A., Olafsdottir E.S. (2019). Ethnopharmacology of the club moss subfamily Huperzioideae (Lycopodiaceae, Lycopodiophyta): A phylogenetic and chemosystematic perspective. J. Ethnopharmacol..

[34] Li G.Y., Su Y.Y. (2017). Determination of Huperzine A content in *Huperzia serrata* from different producing areas by LC-MS method. J. Zhejiang Agric. Sci..

[35] Chen M., Tu Y.S., Ye L.N., Yang B.Y. (2017). Effect of amino acids on thallus growth and Huperzine-a accumulation in *Huperzia serrata*. Chin. Bull. Bot..

[36] Ye L.N., Tu Y.S., Huang Q., Yu X., Yuan H.H., Lu S.M. (2017). Studies on H_2_O_2_ induced effect on *Huperzia serrata* in vitro. Chin. Tradit Med..

[37] Debnath S.C., Goyali J.C. (2020). In vitro propagation and variation of antioxidant properties in micropropagated *Vaccinium* berry plants-a review. Molecular.

[38] White J.D., Li Y., Kim J., Terinek M. (2013). A novel synthesis of (-)-Huperzine A via tandem intramolecular aza-Prins cyclization-cyclobutane fragmentation. Org. Lett..

[39] Zhang J., Liu J., Dai L.F., Zhang H.Y., Chen M.N., Cai X.F., Xie J.K., Luo X.D. (2019). Unlocking the potential antioxidant and anti-inflammatory activities of *Rhododendron molle* G. Don. Pak. J. Pharm. Sci..

[40] Wan A., Ismail N.Z., Omar E.A., Samad N.A., Mohamad S. (2020). GC-MS Evaluation, antioxidant content, and cytotoxic activity of propolis extract from Peninsular Malaysian Stingless Bees, *Tetrigona Apicalis*. J. Evid. Based Complement. Altern. Med..

